# Physical Fitness in Czech Preschool Children: Age-Related Trends Assessed Using the PREFIT Battery

**DOI:** 10.3390/children13091130

**Published:** 2026-08-24

**Authors:** Soňa Jandová, Tomáš Polívka

**Affiliations:** Faculty of Education, Charles University, 116 39 Prague, Czech Republic; sona.jandova@pedf.cuni.cz

**Keywords:** motor competence, early childhood, developmental trends, field-based assessment, muscular strength, motor performance, growth

## Abstract

**Highlights:**

**What are the main findings?**

- Physical fitness was strongly associated with age across all assessed fitness domains in preschool children.
- After adjustment for age and sex, higher BMI SDS was associated with lower handgrip strength, poorer standing long-jump performance, and slower shuttle-run performance.

**What are the implications of the main findings?**

- Both chronological age and age-standardized body-weight status contribute to variability in physical fitness during the preschool years.
- Selected PREFIT tests can be successfully implemented in kindergarten settings and provide useful information about age-related differences in physical fitness.

**Abstract:**

Background: Physical fitness is an important marker of health and development during early childhood. However, data describing fitness characteristics of preschool children from Central and Eastern Europe remain limited. This study examined age-related trends, sex differences, and associations with BMI-for-age z-scores (BMI SDS) in selected components of the PREFIT battery in Czech preschool children. Methods: A cross-sectional study was conducted in 40 children aged 3–6 years (19 girls and 21 boys) attending a public kindergarten in the Czech Republic. Physical fitness was assessed using four selected PREFIT components: handgrip strength, standing long jump, 4 × 10 m shuttle run, and one-leg stance. Associations with age, sex, and BMI SDS were analyzed using linear regression models. Results: Physical fitness was significantly associated with age across all assessed components (all *p* < 0.001). Sex differences were generally small, although boys demonstrated moderately higher handgrip strength than girls. After adjustment for age and sex, higher BMI SDS was associated with lower handgrip strength, poorer standing long-jump performance, and longer shuttle-run times, whereas no significant association was observed for one-leg stance performance. Conclusions: Selected PREFIT components were successfully implemented in a real-world kindergarten setting and were sensitive to age-related differences in physical fitness. Age showed the strongest and most consistent associations with fitness performance, while BMI SDS was independently associated with selected fitness outcomes. Larger multi-site and longitudinal studies are needed to establish population-specific reference values and to clarify the role of early physical fitness in later health and development.

## 1. Introduction

Physical activity (PA) is a key determinant of physical, cognitive, and psychosocial development in early childhood. Evidence suggests that movement behaviors established at this age tend to track into later life and are associated with long-term health outcomes [1,2]. PA is seen as an important activity in promoting health and preventing diseases resulting from contemporary lifestyles. Sedentary lifestyles, characterized by a lack of physical activity together with inappropriate behavior and sleep patterns, are established in early childhood and tend to persist later in life [3]. There is evidence that chronic diseases manifesting in adulthood have their origins in the early years [4]. Lifestyle modification interventions are more difficult in school-aged children and adults than in preschool children. Preschool age thus represents a critical period for the development of health promotion optimization and chronic disease prevention [5]. It is important to create a positive relationship with PA in preschool and school-aged children and to lead them to an active lifestyle that improves health and physical fitness (PF) and persists into adulthood. Individuals with a history of physical activity in childhood tend to be active in adolescence, and active adolescents tend to be more active in adulthood [6]. Preschool age thus represents a key stage of ontogeny for the acquisition of basic motor skills determining the level of motor competence (MC) [7]. At this age, the type of PA and the quality of the movement experience is more important than the intensity and volume of PA [8]. At later stages of an individual’s development, expert studies have demonstrated a positive relationship between MC and PA levels, all health-related fitness factors, including body composition and psychosocial health, and better socioemotional and cognitive skills, self-image, and self-assessment [9,10,11,12,13,14].

Eliminating screen time and sedentary lifestyles in children seems to be a very effective strategy to promote the health of the whole population. A sensitive period for forming a positive relationship with PA is at the ages of 3 and 6–7 years [15].

Previous studies have also demonstrated relationships among physical activity, motor competence, and health-related fitness during childhood [16,17].

Preschool age is characterized by significant changes in motor skill acquisition and maturation of the nervous system [18]. In early childhood, the development of basic motor skills such as locomotion skills and object control skills occurs [17]. Mastery of fundamental movement skills contributes to children’s physical, cognitive, and social development and is essential for integration and current and adult lifestyles [19]. Some studies document a gender difference in the level of basic movement skills [20,21]. Boys excel in different skills from girls, but these differences are not consistent throughout the preschool period [22].

Preschool children with higher physical fitness have been shown to develop better, and the development of specific cognitive skills and motor performance has been shown to occur in parallel [23].

There are not many specific tests that assess the motor competence of preschoolers. A systematic review focusing on physical fitness testing in preschoolers is presented by Ortega [24]. Here, we also find the tests used for preschoolers at the time of publication of the study. It appears that some of the tests used do not have sufficient reliability or validity, and the extent to which they inform about health aspects of preschool children is open to debate. Ortega et al. presented the PREFIT field-based fitness testing battery in preschool children, building on the previously developed ALPHA test battery for children and adolescents aged 6–18 years. The PREFIT test battery consists of a 20 m shuttle-run test for assessing cardiorespiratory fitness, a handgrip-strength and standing long-jump test for assessing musculoskeletal fitness, and a 4 × 10 m shuttle run and one-leg-stance tests for assessing motor fitness, speed/agility, and balance. The PREFIT test battery was described by the authors as an effective tool for assessing the physical fitness of children aged 3–5 years.

Physical fitness tracks important health attributes from childhood and relates to current and future cardiometabolic and functional health. However, in preschool children (3–6 years), feasibility, reliability, and health-related validity of field-based tests have historically been less documented than in school-aged youth. The PREFIT consortium synthesized the scattered evidence and proposed a pragmatic, field-based battery for ages 3–5 years, recommending the 20 m shuttle run for cardiorespiratory fitness, handgrip and standing long jump for musculoskeletal fitness, and 4 × 10 m shuttle run and one-leg stance for motor fitness (speed/agility and balance). This choice was justified by feasibility in real-world preschool settings and continuity with tests widely used from primary school upwards.

From a public-health perspective, early-childhood education (ECE) provides a unique window to lay foundations for movement behaviors and motor competence. Global policy statements continue to highlight the role of physical activity (PA) in healthy growth, bone health, motor and cognitive development, and the need to counter rising physical inactivity levels in children. Population-level calls increasingly emphasize solution-oriented research and implementation in settings with high reach, such as preschools.

Recent studies from Central and Eastern Europe have begun to document physical fitness and motor competence in preschool populations; however, available evidence remains limited and heterogeneous, particularly regarding age-related differences and anthropometric correlates [25].

Despite increasing interest in physical fitness and motor competence during early childhood, evidence from Central and Eastern European countries remains limited. Most available studies have been conducted in Western Europe, North America, or Asia, while relatively few investigations have examined age-related fitness patterns and anthropometric correlates in preschool populations from the Central European region. Recent findings have highlighted the importance of establishing population-specific reference data, because physical fitness and growth characteristics may vary across countries and educational environments. In addition, evidence concerning the relationship between body-weight status and fitness performance during the preschool years remains inconsistent, particularly in samples from Central Europe. Therefore, further research is needed to characterize physical fitness in this age group and to clarify the relative contribution of age, sex, and body composition to fitness performance.

Thus, the present study aimed to examine age-related trends, sex differences, and BMI-related associations in selected PREFIT components in a Czech preschool sample. A secondary aim was to evaluate the implementation of selected PREFIT tests within a real-world kindergarten setting.

## 2. Materials and Methods

### 2.1. Subjects

Participants were 40 preschool children (19 girls and 21 boys) aged 3–6 years attending a public kindergarten in the Czech Republic. All children were reportedly healthy, without any known conditions that would limit normal participation in physical activity, and all were able to follow simple motor instructions required for the fitness assessments. Children with medical conditions limiting participation in physical activity were not eligible. No participants meeting the inclusion criteria were excluded from the statistical analyses.

Recruitment occurred through the kindergarten, and written informed consent was obtained from parents or legal guardians. All procedures were approved by the Ethics Committee of the Department of Physical Education, Faculty of Education, Charles University (approval 3/2024).

### 2.2. Sample-Size Estimation and Justification

Calculation was performed using the G*Power software (version 3.1; Heinrich Heine University Düsseldorf, Düsseldorf, Germany). The effect size was determined to be moderate (f^2^ = 0.2) based on conventional criteria and in light of previous research on physical activity in children. The level of statistical significance was set at α = 0.05 and the required statistical power at 0.75. The model included one independent variable (age).

All input parameters were specified in advance to ensure transparency and reproducibility of the calculation. Based on these parameters, the required sample size was estimated at *n* = 37 participants.

### 2.3. Procedures

Data collection was conducted during regular morning school hours in familiar indoor and outdoor play areas to ensure that children were assessed in an environment consistent with their daily routine. Testing took place over a single session for each group of children and was administered by trained assessors experienced in working with the preschool population. All children completed anthropometric measurements first, followed by the fitness tests. Assessors demonstrated each task, allowed brief practice trials when needed, and provided standardized verbal encouragement while avoiding competitive pressure. Children were tested individually or in small groups depending on the nature of each fitness task.

Parents completed a short questionnaire reporting their child’s typical weekly physical activity outside of kindergarten, including time spent in unstructured active play and participation in organized sport. These variables were not included in inferential analyses and are presented solely for descriptive context.

### 2.4. Anthropometry Measurements

Height was measured to the nearest 0.1 cm using a portable stadiometer, and body mass to the nearest 0.1 kg using a calibrated digital scale. Waist circumference was measured at the midpoint between the lower rib and the iliac crest using a nonelastic tape.

BMI (kg·m^−2^) was calculated from measured height and body mass. Age- and sex-specific BMI percentiles and BMI-for-age z-scores (BMI SDS) were calculated using WHO growth references. BMI SDS values were used to describe weight status within the sample and to classify children as underweight, normal weight, overweight, or obese according to WHO criteria.

### 2.5. Physical Fitness Assessments

Four components of the PREFIT test battery were used, reflecting muscular strength, lower-body power, speed–agility, and balance, all appropriate for preschool children:

- Handgrip strength (kg): Measured using a child-appropriate dynamometer. Children performed the test with their dominant hand in a standing position, and the best of two attempts was recorded.
- Standing long jump (cm): Used to assess lower-body power. Children jumped from a standing position with feet shoulder-width apart, and the longest of two jumps was recorded.
- 4 × 10 m shuttle run (s): Assessed speed–agility. The test was performed without the use of sponges to reduce cognitive load for younger children. Time was recorded with a digital stopwatch.
- One-leg stance (s): Evaluated static balance. Children stood on their preferred leg with the opposite foot lifted slightly off the ground and eyes open. Time to loss of balance or foot contact with the ground was recorded, up to a preset maximum.

All fitness tests were administered following standardized instructions, and testing order was structured to minimize fatigue.

### 2.6. Statistical Analysis

Descriptive statistics (means and standard deviations) were calculated separately for girls and boys for all anthropometric and fitness variables. Between-sex differences were examined using Welch’s *t*-tests. In addition to statistical significance, effect sizes were calculated using Hedges’ g and interpreted according to conventional criteria.

To examine associations between age and physical fitness, separate linear regression models were estimated for each fitness outcome. In Model 1, fitness performance was regressed on age and sex. Regression coefficients (β), standard error (SE), 95% confidence intervals (CIs), and adjusted coefficients of determination (R^2^adj) are reported.

To examine associations between BMI and physical fitness, a second set of models was estimated. In Model 2, BMI, age, and sex were entered simultaneously as predictors in order to evaluate the relationship between BMI and fitness performance while controlling for potential confounding effects of age and sex. Additional descriptive analyses were performed using BMI-for-age z-scores (BMI SDS) derived from WHO growth references.

All analyses were performed using IBM SPSS Statistics, version 31. Owing to the modest sample size and relatively wide age range, findings were interpreted cautiously. Statistical significance was set at *p* < 0.05.

## 3. Results

A total of 40 preschool children (girls: *n* = 19; boys: *n* = 21) completed all anthropometric and PREFIT fitness assessments. Descriptive characteristics of the sample are summarized in Table 1. Girls and boys did not differ significantly in age (5.18 ± 0.99 vs. 5.30 ± 0.93 years) or height (113.27 ± 7.67 vs. 116.68 ± 7.55 cm), whereas boys had higher body mass and waist circumference, resulting in a higher mean BMI (16.57 ± 3.41 vs. 14.89 ± 1.24 kg·m^2^). Based on WHO BMI-for-age z-scores (BMI SDS), five children (four boys and one girl) were classified as overweight and two boys met the criterion for obesity.

### 3.1. Sex Differences in Physical Fitness

Between-sex comparisons of PREFIT components are presented in Table 2. Boys demonstrated significantly higher handgrip strength than girls (7.14 ± 2.01 kg vs. 5.77 ± 1.84 kg; Welch *t* = −2.26, *p* = 0.0296), with a moderate effect size (Hedges’ *g* = 0.70), indicating a moderate sex-related difference in upper-limb strength. No significant sex differences were observed for the remaining tests. For the 4 × 10 m shuttle run, a moderate effect size was observed in favor of boys (Hedges’ *g* = −0.57), although the difference did not reach statistical significance (*t* = 1.84, *p* = 0.0734). This result indicates a moderate between-group difference accompanied by substantial statistical uncertainty and should therefore be interpreted cautiously.

**Figure 1:**
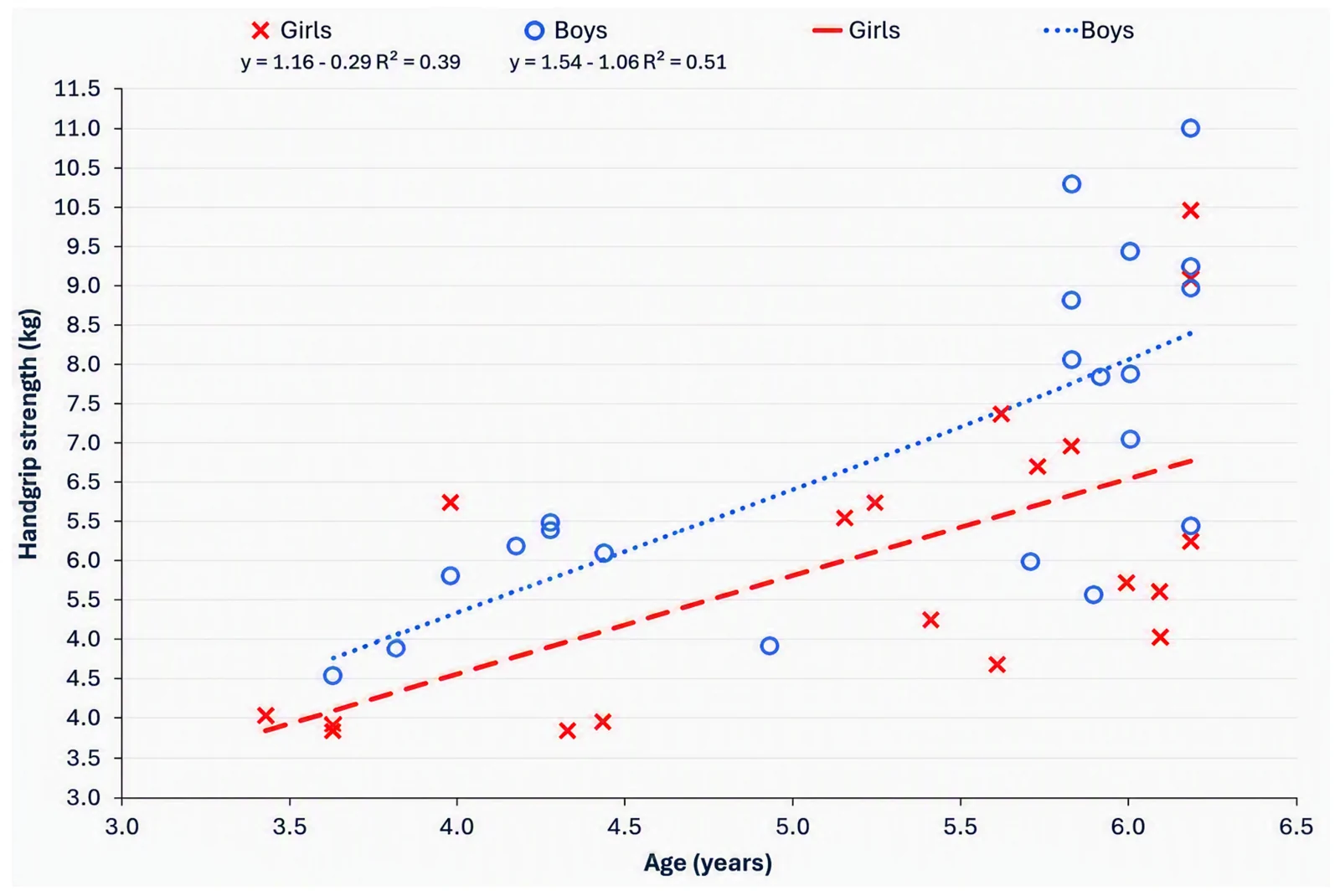
Handgrip strength by age and sex. Points are individual observations, solid lines are within-sex linear fits, colored lines represent sex-specific linear regressions, and the dashed line represents the pooled regression for the total sample.

**Figure 2:**
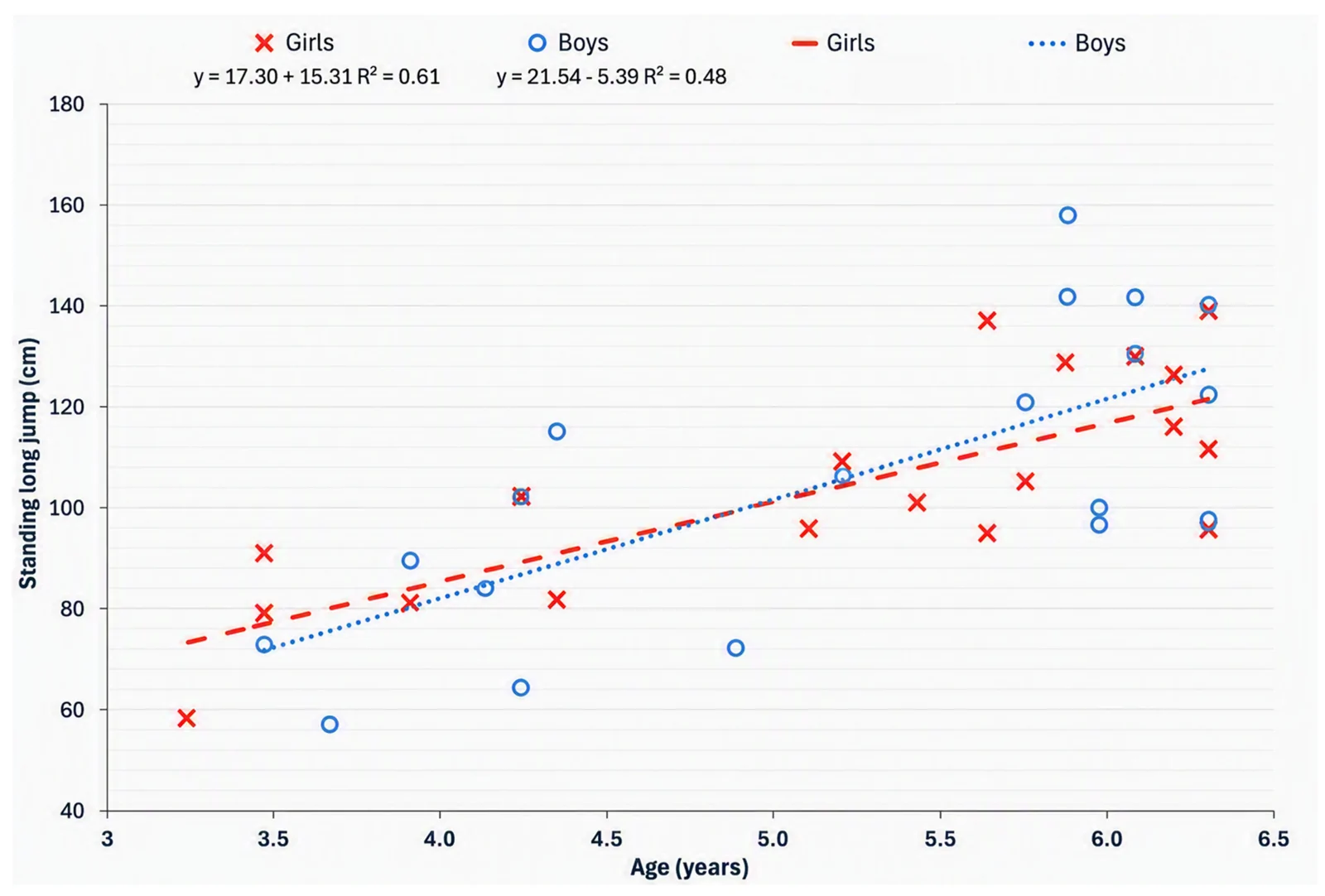
Standing long jump by age and sex. Points are individual observations, colored lines represent sex-specific linear regressions, and the dashed line represents the pooled regression for the total sample.

**Figure 3:**
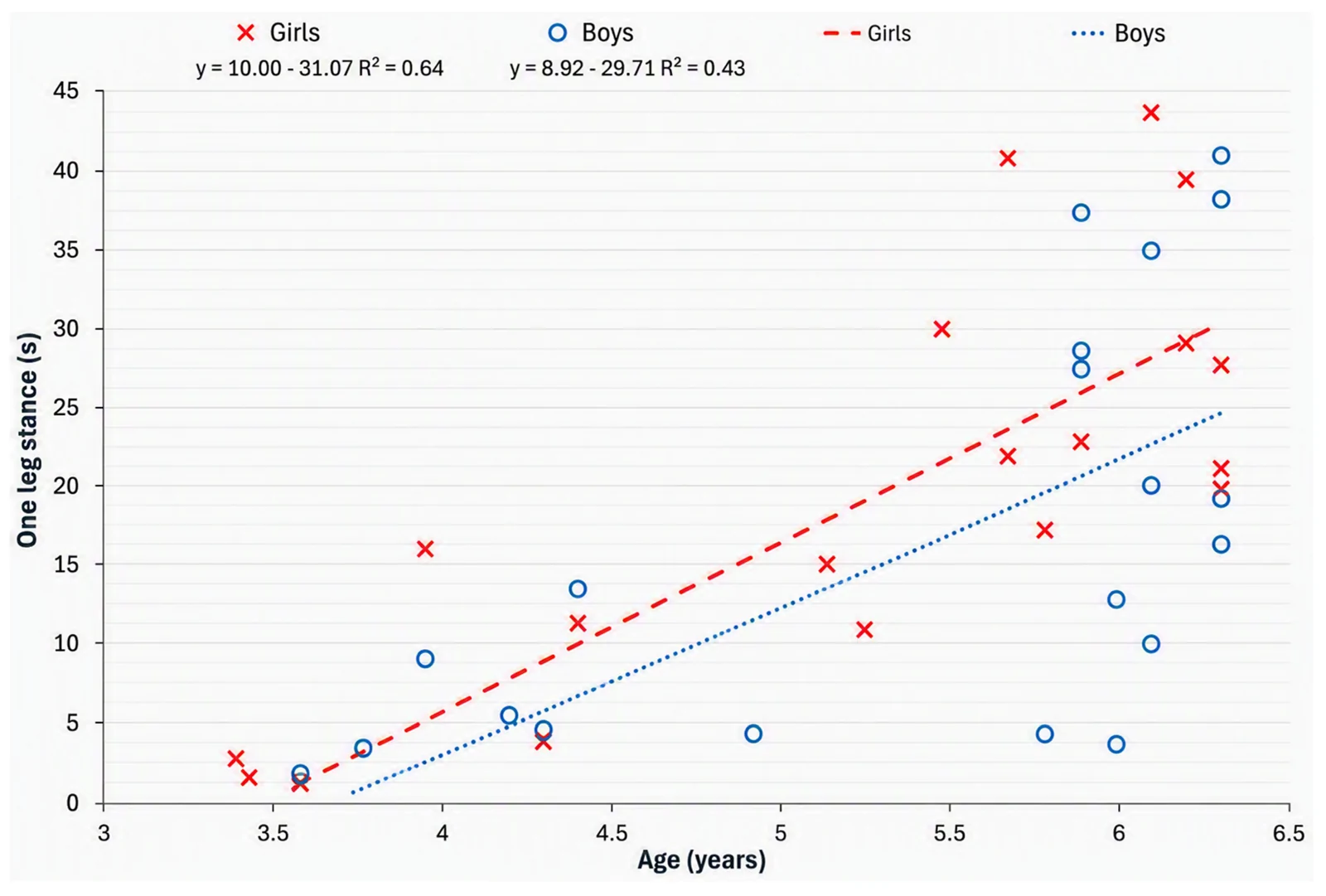
One-leg stance time by age and sex. Points are individual observations, colored lines represent sex-specific linear regressions, and the dashed line represents the pooled regression for the total sample.

**Figure 4:**
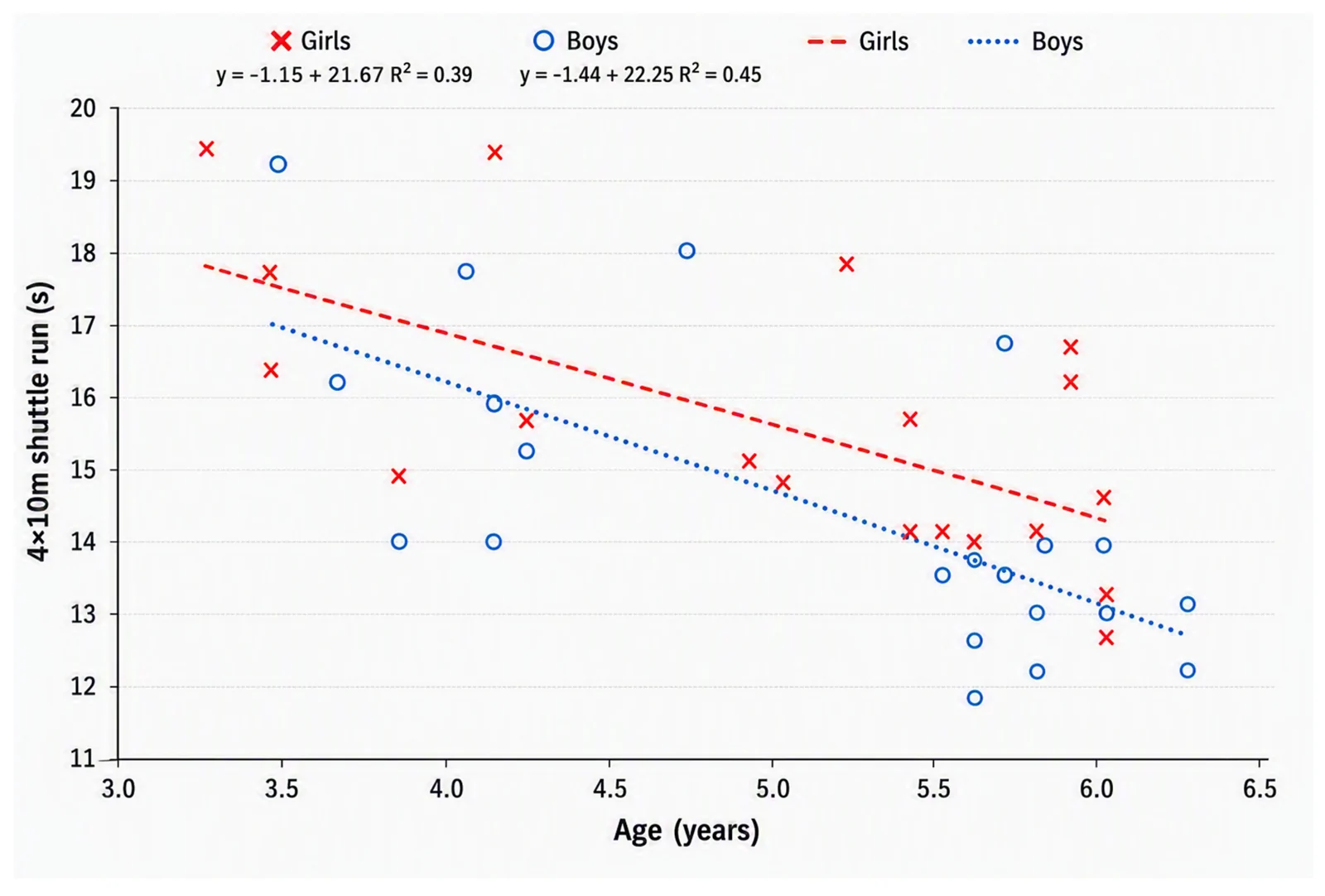
4 × 10 m shuttle-run time by age and sex. Points are individual observations, solid lines are within-sex linear fits, colored lines represent sex-specific linear regressions, and the dashed line represents the pooled regression for the total sample.

Effect sizes for standing long jump (*g* = 0.14) and one-leg stance (*g* = −0.26) were small, indicating largely overlapping performance distributions between girls and boys despite minor differences in point estimates. These findings highlight substantial interindividual variability within both sexes, as illustrated in Figure 1, Figure 2, Figure 3 and Figure 4.

### 3.2. Age-Related Trends in Physical Fitness

Age was strongly associated with performance across all selected PREFIT components (Table 3). In regression models adjusted for sex, each additional year of age was associated with higher handgrip strength (β = 1.40 kg·year^−1^, *p* < 0.001), longer standing long-jump distance (β = 19.47 cm·year^−1^, *p* < 0.001), and better one-leg stance performance (β = 9.33 s ·year^−1^, *p* < 0.001), as well as faster shuttle-run performance (β = −1.33 s·year^−1^, *p* < 0.001). Older children also completed the 4 × 10 m shuttle run in less time (β = −1.33 s·year^−1^, *p* < 0.001), indicating higher speed–agility performance.

Adjusted coefficients of determination ranged from 0.392 to 0.513, suggesting moderate-to-strong age-related associations across all fitness outcomes. These relationships are illustrated in Figure 1, Figure 2, Figure 3 and Figure 4, and demonstrate a consistent pattern of better performance among older preschool children.

### 3.3. Associations Between BMI and Fitness

Associations between BMI and physical fitness were examined using regression models adjusted for both age and sex (Table 4). After controlling for these variables, higher BMI was significantly associated with lower handgrip strength and shorter standing long-jump distance. Similarly, higher BMI was associated with longer 4 × 10 m shuttle-run times, indicating lower speed–agility performance. In contrast, no significant association was observed between BMI and one-leg stance performance. Adjusted coefficients of determination indicated that age, sex, and BMI together explained a substantial proportion of variance in physical fitness outcomes.

### 3.4. Overall Interpretation and Integration of Findings

Overall, the results indicate limited evidence of sex differences in physical fitness, with the exception of a moderate effect favoring boys in handgrip strength. Chronological age was consistently associated with all fitness outcomes and showed the strongest relationships across models. In addition, BMI demonstrated independent associations with muscular strength, lower-body power, and speed–agility performance after adjustment for age and sex. Collectively, these findings suggest that both age and body-size characteristics contribute to variation in physical fitness during the preschool years, although age-related associations were generally stronger and more consistent.

## 4. Discussion

### 4.1. Principal Findings

This study examined age-related trends, sex differences, and associations between BMI-for-age z-scores (BMI SDS) and selected PREFIT components in a Czech preschool sample. The main findings indicate that (i) physical fitness was strongly associated with chronological age across all assessed domains, (ii) sex differences were generally small, although boys demonstrated moderately higher handgrip strength than girls, and (iii) BMI SDS was independently associated with several fitness outcomes after adjustment for age and sex.

### 4.2. Age-Related Trends in Physical Fitness

The strong age-related associations observed across all selected PREFIT components are fully consistent with developmental and empirical research. Early childhood is characterized by rapid improvements in motor coordination, neuromuscular control, and movement efficiency, which are reflected in better performance in strength-, power-, agility-, and balance-related tasks [7,17].

Similarly, both cross-sectional and longitudinal studies have reported positive age gradients in physical fitness and motor competence among preschool children. Ortega et al. highlighted that the PREFIT tests are sensitive to age-related differences across the preschool years, particularly for muscular strength and motor competence. Likewise, Tanaka et al. reported better performance in several fitness and motor tasks with increasing age in Japanese preschool children [18,24].

In the present study, age explained a substantial proportion of variance in fitness performance (adjusted R^2^ ranging from approximately 0.39 to 0.51). These findings indicate that chronological age is one of the strongest correlates of physical fitness during early childhood. Nevertheless, because the study was cross-sectional, the observed relationships should be interpreted as age-related associations rather than direct evidence of maturational or developmental change.

Importantly, these results support the recommendation that fitness outcomes in preschool populations should be interpreted relative to age and developmental stage rather than viewed as stable indicators of individual ability.

### 4.3. Sex Differences in Preschool Physical Fitness

In contrast to adolescence, where sex differences in physical fitness are more pronounced, the present study found only limited evidence of sex-related differences. Boys demonstrated moderately higher handgrip strength than girls, whereas differences in standing long jump, shuttle-run performance, and balance were small and statistically non-significant.

This pattern is consistent with previous research suggesting that sex differences in preschool fitness are generally task-specific and relatively modest before puberty [20,21]. Similarly, Kokstejn et al. reported that sex differences in motor competence are not stable across early childhood and may vary according to the specific skill assessed [22].

The moderate effect observed for handgrip strength may reflect differences in movement behaviors, play preferences, or early neuromuscular characteristics. However, the relatively small sample, and wide variability within both groups warrant cautious interpretation. Therefore, while the present findings suggest stronger age-related than sex-related associations with physical fitness, they do not support firm conclusions regarding the usefulness or irrelevance of sex-specific normative values.

### 4.4. BMI and Physical Fitness

One of the most important findings of the present study was that BMI-for-age z-scores (BMI SDS) were significantly associated with several fitness outcomes after adjustment for age and sex. Specifically, higher BMI SDS was associated with lower handgrip strength, poorer standing long-jump performance, and slower shuttle-run performance, whereas no significant association was observed for one-leg stance performance. These findings indicate that age- and sex-standardized body-weight status may contribute to variability in physical fitness already during the preschool years.

These findings demonstrate the importance of considering age and sex when examining associations between BMI SDS and physical fitness in preschool populations. The fact that significant relationships remained evident after age standardization strengthens the interpretation that body-weight status itself may contribute to selected aspects of motor performance.

The observed negative associations between BMI and standing long-jump and shuttle-run performance are consistent with previous studies showing that higher body mass may represent a biomechanical disadvantage in activities requiring acceleration, propulsion of body mass, or rapid changes in direction [11].

The negative association between BMI and handgrip strength was less expected. One possible explanation is that BMI may not accurately distinguish between fat mass and lean mass in young children. Therefore, higher BMI in this cohort may primarily reflect increases in adiposity rather than muscularity, resulting in lower relative functional performance.

Interestingly, no association was observed between BMI and one-leg stance performance. This may suggest that static balance at this age is influenced more strongly by neuromotor maturation, sensory integration, and task familiarity than by body-size characteristics. Similar inconsistencies have been reported in studies examining relationships between anthropometry and motor competence in preschool children [12,17].

Overall, these findings indicate that BMI SDS may already influence selected aspects of physical fitness during the preschool years, particularly tasks involving power and speed–agility. However, the magnitude of these associations remained smaller than those observed for chronological age and should be interpreted cautiously given the limited sample size and cross-sectional design.

### 4.5. Integration Within the PREFIT Framework

The present findings support the usefulness of selected PREFIT components as indicators of age-related differences in physical fitness. Consistent with the PREFIT framework proposed by Ortega et al., the tests used in the present study were sensitive to differences associated with age and capable of discriminating between children with different performance levels [24].

At the same time, our results highlight that associations with BMI SDS may be stronger than originally assumed in preschool populations when age and sex are appropriately controlled. Therefore, selected PREFIT tests appear suitable not only for describing age-related patterns but also for examining relationships between age-standardized body-weight status and functional motor performance.

### 4.6. Strengths and Limitations

A key strength of the present study is the use of a standardized field-based protocol aligned with selected components of the PREFIT battery, allowing comparison with the international literature. In addition, testing was conducted in a real-world kindergarten environment, enhancing ecological validity and demonstrating that the selected assessments can be successfully implemented in preschool settings.

However, several limitations should be acknowledged. The study was conducted in a single kindergarten with a relatively small sample, limiting statistical power and the generalizability of the findings, and therefore should not be considered representative of Czech preschool children as a whole. The absence of cardiorespiratory fitness assessment (20 m shuttle run) restricts interpretation across the full spectrum of PREFIT-related fitness domains. Manual timing in the shuttle run may also have introduced measurement error. Furthermore, physical activity data were based on parental reports and were not included in the inferential analyses. Finally, because of the cross-sectional design, observed associations should not be interpreted as evidence of developmental change or causal relationships.

### 4.7. Implications for Practice and Future Research

From a practical perspective, the findings support the integration of structured fitness assessments into early-childhood education settings. The observed age-related associations suggest that selected PREFIT tests may provide useful information about fitness differences across preschool-age groups and may help practitioners identify children who could benefit from additional opportunities for physical activity and motor development.

For research, larger multi-site cohorts are needed to establish national reference values and improve the generalizability of findings. Future studies should employ longitudinal designs to determine how physical fitness develops across early childhood and whether early fitness levels predict subsequent health, motor competence, and educational outcomes. The inclusion of objective physical activity measures, such as accelerometry, would also strengthen future investigations.

## 5. Conclusions

The present study demonstrates that selected components of the PREFIT battery can be successfully implemented in a real-world kindergarten setting and are sensitive to age-related differences in physical fitness among preschool children. Significant associations between age and performance were observed across all assessed fitness domains, indicating that chronological age is an important correlate of physical fitness during early childhood.

Sex differences were generally small, although boys demonstrated moderately higher handgrip strength than girls. After adjustment for age and sex, BMI SDS was significantly associated with handgrip strength, standing long-jump performance, and shuttle-run performance, suggesting that age-standardized body-weight status may contribute to variability in selected fitness outcomes even during the preschool years.

The findings support the use of selected PREFIT tests for describing age-related differences in physical fitness in early childhood. However, given the cross-sectional design and limited sample size, conclusions regarding developmental processes and population-level patterns should be interpreted cautiously.

Future research based on larger multi-site cohorts and longitudinal designs is needed to establish population-specific reference values and to better understand how early physical fitness relates to subsequent health, motor competence, and developmental outcomes.

## Figures and Tables

**Table 1:** Anthropometric characteristics by sex (mean ± SD).

Variable	Girls, *n* = 19 (Mean ± SD)	Boys, *n* = 21 (Mean ± SD)
Age (years)	5.18 ± 0.99	5.30 ± 0.93
Height (cm)	113.27 ± 7.67	116.68 ± 7.55
Weight (kg)	19.25 ± 3.16	23.07 ± 6.61
Waist (cm)	53.06 ± 4.59	58.71 ± 7.44
BMI (kg/m^2^)	14.89 ± 1.24	16.57 ± 3.41

**Table 2:** PREFIT outcomes by sex with Welch *t*-test on raw values.

Test	Girls *n* = 19 (Mean ± SD)	Boys *n* = 21 (Mean ± SD)	*t*	*p*	Effect Size (Hedges’ *g*)
Handgrip (kg)	5.77 ± 1.84	7.14 ± 2.01	−2.26	0.0296	0.7
Standing long jump (cm)	105.03 ± 21.66	108.71 ± 28.85	−0.46	0.6493	0.14
4 × 10 m shuttle run (s)	15.70 ± 1.80	14.59 ± 2.01	1.84	0.0734	−0.57
One-leg stance (s)	20.79 ± 12.29	17.54 ± 12.59	0.82	0.4153	−0.26

Note: Effect size is reported as Hedges’ *g* (boys − girls). Positive values indicate higher performance in boys; negative values indicate higher performance in girls.

**Table 3:** Age-trend linear regressions (pooled; adjusted for sex). β is per 1 year of age.

Test	β_Age	SE	95% CI	*p*	R^2^_adj
Handgrip (kg)	1.40	0.26	(0.86, 1.86)	<0.001	0.409
Standing long jump (cm)	19.47	3.00	(13.25, 25.59)	<0.001	0.513
4 × 10 shuttle run (s)	−1.33	0.26	(−1.81, −0.79)	<0.001	0.392
One-leg stance (s)	9.33	1.49	(6.50, 12.44)	<0.001	0.495

**Table 4:** BMI-trend linear regressions (pooled; adjusted for sex). β is per 1 kg·m^−2^ of BMI.

Test	β_BMI	SE	95% CI	*p*	R^2^_adj
Handgrip (kg)	−0.594	0.196	(−0.993, −0.196)	0.005	0.583
Standing long jump (cm)	−5.910	2.550	(−11.10, −0.739)	0.026	0.553
4 × 10 shuttle run (s)	0.676	0.194	(0.283, 1.068)	0.001	0.570
One-leg stance (s)	−2.020	1.270	(−4.600, 0.558)	0.121	0.532

Note: BMI SDS-motor performance linear regressions (pooled; adjusted for age and sex). β coefficients represent the expected change in outcome for a one-unit increase in BMI SDS. Positive β values indicate higher outcome scores with increasing BMI SDS, whereas negative β values indicate lower outcome scores.

## Data Availability

The data presented in this study are available at https://docs.google.com/spreadsheets/d/1t5rbP9YtxM1reSsusOLCqU0vHDyHKCdJsM3040bigSk/edit?gid=0#gid=0 (accessed on 10 July 2026).

## References

[1] Carson V., Lee E.-Y., Hewitt L., Jennings C., Hunter S., Kuzik N., Stearns J.A., Unrau S.P., Poitras V.J., Gray C. (2017). Systematic review of the relationships between physical activity and health indicators in the early years (0–4 years). BMC Public Health.

[2] Veldman S.L.C., Chin APaw M.J.M., Altenburg T.M. (2021). Physical activity and prospective associations with indicators of health and development in children aged <5 years: A systematic review. Int. J. Behav. Nutr. Phys. Act..

[3] Ding D., Lawson K.D., Kolbe-Alexander T.L., Finkelstein E.A., Katzmarzyk P.T., van Mechelen W., Pratt M. (2016). The economic burden of physical inactivity: A global analysis of major non-communicable diseases. Lancet.

[4] Berenson G.S., Srinivasan S.R., Bao W., Newman W.P., Tracy R.E., Wattigney W.A. (1998). Association between Multiple Cardiovascular Risk Factors and Atherosclerosis in Children and Young Adults. N. Engl. J. Med..

[5] Goldfield G.S., Harvey A., Grattan K., Adamo K.B. (2012). Physical Activity Promotion in the Preschool Years: A Critical Period to Intervene. Int. J. Environ. Res. Public Health.

[6] Malina R.M. (2001). Adherence to Physical Activity from Childhood to Adulthood: A Perspective from Tracking Studies. Quest.

[7] Hulteen R.M., Morgan P.J., Barnett L.M., Stodden D.F., Lubans D.R. (2018). Development of Foundational Movement Skills: A Conceptual Model for Physical Activity Across the Lifespan. Sports Med..

[8] Schmutz E.A., Leeger-Aschmann C.S., Kakebeeke T.H., Zysset A.E., Messerli-Bürgy N., Stülb K., Arhab A., Meyer A.H., Munsch S., Puder J.J. (2020). Motor Competence and Physical Activity in Early Childhood: Stability and Relationship. Front. Public Health.

[9] Bremer E., Cairney J. (2016). Fundamental Movement Skills and Health-Related Outcomes: A Narrative Review of Longitudinal and Intervention Studies Targeting Typically Developing Children. Am. J. Lifestyle Med..

[10] Luz C., Cordovil R., Rodrigues L.P., Gao Z., Goodway J.D., Sacko R.S., Nesbitt D.R., Ferkel R.C., True L.K., Stodden D.F. (2019). Motor competence and health-related fitness in children: A cross-cultural comparison between Portugal and the United States. J. Sport Health Sci..

[11] D’Hondt E., Deforche B., Gentier I., Verstuyf J., Vaeyens R., De Bourdeaudhuij I., Philippaerts R., Lenoir M. (2014). A longitudinal study of gross motor coordination and weight status in children: Gross Motor Coordination and Weight Status. Obesity.

[12] Timmons B.W., LeBlanc A.G., Carson V., Connor Gorber S., Dillman C., Janssen I., Kho M.E., Spence J.C., Stearns J.A., Tremblay M.S. (2012). Systematic review of physical activity and health in the early years (aged 0–4 years). Appl. Physiol. Nutr. Metab..

[13] Leonard H.C. (2016). The Impact of Poor Motor Skills on Perceptual, Social and Cognitive Development: The Case of Developmental Coordination Disorder. Front. Psychol..

[14] Vedul-Kjelsås V., Sigmundsson H., Stensdotter A.-K., Haga M. (2011). The relationship between motor competence, physical fitness and self-perception in children. Child Care Health Dev..

[15] Gustafson S.L., Rhodes R.E. (2006). Parental Correlates of Physical Activity in Children and Early Adolescents. Sports Med..

[16] Fisher A., Reilly J.J., Kelly L.A., Montgomery C., Williamson A., Paton J.Y., Grant S. (2005). Fundamental Movement Skills and Habitual Physical Activity in Young Children. Med. Sci. Sports Exerc..

[17] Stodden D.F., Goodway J.D., Langendorfer S.J., Roberton M.A., Rudisill M.E., Garcia C., Garcia L. (2008). A Developmental Perspective on the Role of Motor Skill Competence in Physical Activity: An Emergent Relationship. Quest.

[18] Tanaka C., Hikihara Y., Ohkawara K., Tanaka S. (2012). Locomotive and Non-Locomotive Activity as Determined by Triaxial Accelerometry and Physical Fitness in Japanese Preschool Children. Pediatr. Exerc. Sci..

[19] Lubans D.R., Morgan P.J., Cliff D.P., Barnett L.M., Okely A.D. (2010). Fundamental Movement Skills in Children and Adolescents: Review of Associated Health Benefits. Sports Med..

[20] Foulkes J.D., Knowles Z., Fairclough S.J., Stratton G., O’Dwyer M., Ridgers N.D., Foweather L. (2015). Fundamental Movement Skills of Preschool Children in Northwest England. Percept. Mot. Ski..

[21] Latorre-Román P.Á., Mora-López D., García-Pinillos F. (2016). Intellectual maturity and physical fitness in preschool children. Pediatr. Int..

[22] Kokštejn J., Musálek M., Tufano J.J. (2018). Construct Validity of the Movement Assessment Battery for Children-Second Edition Test in Preschool Children with Respect to Age and Gender. Front. Pediatr..

[23] Hillman C.H., Buck S.M., Themanson J.R., Pontifex M.B., Castelli D.M. (2009). Aerobic fitness and cognitive development: Event-related brain potential and task performance indices of executive control in preadolescent children. Dev. Psychol..

[24] Ortega F.B., Cadenas-Sánchez C., Sánchez-Delgado G., Mora-González J., Martínez-Téllez B., Artero E.G., Castro-Piñero J., Labayen I., Chillón P., Löf M. (2014). Systematic Review and Proposal of a Field-Based Physical Fitness-Test Battery in Preschool Children: The PREFIT Battery. Sports Med..

[25] Saczuk J., Wasiluk A., Chaliburda I. (2024). Physical Fitness and Motor Skills of Five-Year-Olds with Different Weight-Height Proportions. Pol. J. Sport Tour..

